# The Effects of Dietary *Bacillus amyloliquefaciens* TL106 Supplementation, as an Alternative to Antibiotics, on Growth Performance, Intestinal Immunity, Epithelial Barrier Integrity, and Intestinal Microbiota in Broilers

**DOI:** 10.3390/ani12223085

**Published:** 2022-11-09

**Authors:** Chengling Bao, Wenxiu Zhang, Jian Wang, Yajing Liu, Heng Cao, Feiyu Li, Suozhu Liu, Zhengda Shang, Yunhe Cao, Bing Dong

**Affiliations:** 1 State Key Laboratory of Animal Nutrition, College of Animal Science and Technology, China Agricultural University, Beijing 100193, China; 2 College of Animal Science, Tibet Agricultural and Animal Husbandry University, Linzhi 860000, China

**Keywords:** *Bacillus amyloliquefaciens*, barrier function, gut microbiota, immunity, broilers

## Abstract

**Simple Summary:**

Global demand for chicken is increasing. Antibiotics are widely added to feed because of their ability to prevent zoonotic diseases and improve the growth performance of livestock. However, because of problems of food safety and environmental pollution caused by antibiotics, there is an increasing demand to develop and apply antibiotic alternatives in feed. Probiotics are one of the ideal alternatives to antibiotics. The *Bacillus amyloliquefaciens* is a common and healthy probiotic. Our previous studies have shown that *Bacillus amyloliquefaciens* TL016 has good probiotic effects in animals and also has the potential to be an alternative to in-feed antibiotic. In this study, different levels of *Bacillus amyloliquefaciens* were added to broiler diets to investigate the probiotic mechanism of *Bacillus amyloliquefaciens* on broilers. The results indicate that dietary TL106 improves the growth performance, immune response ability, and intestinal health of broilers and regulates the development and intestinal microbiota composition of broilers. *Bacillus amyloliquefaciens* TL106 may be a suitable alternative to feeding antibiotics to improve the health and performance of broilers.

**Abstract:**

A total of 240 1-day-old Arbor Acres male broilers were randomly divided into five dietary treatments (control feed (CON), supplemented with 75 mg/kg aureomycin (ANT), supplemented with 7.5 × 10^8^ CFU/kg (Ba1) and 2.5 × 10^9^ CFU/kg (Ba1), and 7.5 × 10^9^ CFU/kg (Ba3) *Bacillus amyloliquefaciens* TL106, respectively) to investigate the probiotic effect of TL106 instead of antibiotics in broilers. On days 1–21, the average daily gain of broilers in the Ba groups was increased compared with the CON group (*p* < 0.05). In addition, the feed/gain ratio of broilers in the Ba groups was lower than that of broilers in the CON and ANT groups on days 22–42 and days 1–42 (*p* < 0.05). Compared with the CON group, dietary TL106 increased the digestibility of crude fiber and crude protein (*p* < 0.05), and the effect was similar to that of the ANT group. The levels of IL-1β, IFN-γ, and IL-6 in serum, jejunum, and ileum of broilers fed TL106 were decreased compared with the control group (*p* < 0.05). The mRNA expression of tight junction proteins in broilers of ANT and Ba groups was higher than the control group (*p* < 0.05). After 21 days, villus height and the ratio of villus height to crypt depth of duodenum and jejunum of broilers fed TL106 were higher than the control group (*p* < 0.05). The concentrations of short-chain fatty acids such as lactate, acetate, propionate, and butyrate in cecal digesta of broilers dietary TL106 were higher than the control group (*p* < 0.05). The supplementation with TL106 altered the compositions and diversity of the cecal microbiota of broilers. Moreover, supplementation with TL106 improved the ratio of *Firmicutes* to *Bacteroidetes* and decreased the relative abundance of *Proteobacteria* on days 21 and 28, while the abundance of *Peptostreptococcaceae*, *Ruminococcaceae* and *Lactobacillaceae* was increased. On days 35 and 42, broilers fed TL106 had an increased total abundance of *Firmicutes* and *Bacteroidetes* and decreased abundances of *Lactobacillaceae*, while the abundance of *Barnesiellaceae* was increased. In conclusion, dietary supplementation with TL106 improved the broiler’s growth performance, immune response capacity, gut health, modulated development, and composition of the gut microbiota in broilers. It is suggested that *Bacillus amyloliquefaciens* TL106 may be a suitable alternative to in-feed antibiotics to improve broiler health and performance.

## 1. Introduction

Chicken has played an important role in satisfying people’s availability of adequate energy, vitamins, and proteins. The global demand for chicken is increasing considerably [1]. To prevent and treat infectious diseases and improve the growth performance of livestock, antibiotics have been widely supplemented to feed. However, the high-dose use of antibiotics in animals has caused food safety concerns and environmental pollution and resulted in enhanced drug resistance of bacteria [2]. Currently, an increasing number of countries have implemented measures to eliminate the use of antibiotics as growth promoters at subtherapeutic levels in livestock production [3]. Therefore, there is an increasing need for the development and application of antibiotic alternatives in feed.

Because of the many characteristics of probiotics, such as non-toxic side effects, nondrug resistance, no residues, low cost, and significant effects in promoting growth and preventing and treating diseases, probiotics have become one of the ideal alternatives to antibiotics [4]. Previous studies have shown that probiotics have beneficial effects on growth performance, feed conversion, and the immune response of livestock and poultry [5,6]. Currently, the probiotics used for animal production mainly include *Bacillus*, *Bifidobacterium*, *Lactobacillus*, *Streptococcus faecalis*, *Actinomyces*, and yeast. Among the numerous probiotics, *Bacillus* spp. (including *Bacillus subtilis*, *Bacillus amyloliquefaciens*, *Bacillus coagulans*, and *Bacillus licheniformis*, etc.) was considered to be the most promising because the spores produced by *Bacillus* are highly resistant to the harsh conditions of the host digestive tract, and *Bacillus* can produce a variety of digestive enzymes and substances with bacteriostatic activity [7,8]. Previous studies indicated that dietary supplementation of *Bacillus amyloliquefaciens* (*B. amyloliquefaciens*) improved the intestinal microflora of broilers, increased intestinal development and the body’s immunity, and improved growth performance [9,10,11]. It has been reported that *B. amyloliquefaciens* has potential beneficial effects on growth performance, disease resistance, and intestinal health of broilers and is a good alternative to antibiotics in broiler diets [12,13].

To date, several studies have shown that one of the main mechanisms by which probiotics exert their beneficial effects in poultry is by increasing digestion and absorption of nutrients [14]. Probiotics can affect villus height (VH) and crypt depth (CD) of the small intestine, promote the secretion of digestive enzymes and improve the integrity of the intestinal epithelium, thereby improving digestion and absorption efficiency of nutrients [15,16]. Probiotics affect the host’s immune function via a variety of pathways. For instance, in terms of non-specific immunity, probiotics can increase the secretion of mucus, inhibit the growth of proliferation of pathogenic microorganisms, reduce intestinal permeability, activate macrophages and their phagocytic capacity, as well as the activity of natural killer cells. In terms of specific immunity, it has the effect of increasing the secretion of antibodies IgA, IgM, and IgG and influencing pathways of the immune system at all levels through cytokines and other regulatory factors [17,18]. The study by Luan et al. [11] showed that *B. amyloliquefaciens* could increase the serum IgA, IgM, and IgG levels in broilers, which in turn enhanced the immunity of broilers. In addition, several studies have indicated that dietary probiotics in poultry can increase the abundance of intestinal beneficial bacteria as well as maintain the function of host intestinal microbiota [19], thereby achieving intestinal homeostasis and acting as the first line of defense against pathogenic bacteria [20,21]. Wang et al. [12] suggested that dietary supplementation with *B. amyloliquefaciens* in broiler could modulate the intestinal microbiota. Probiotics convert nutrients in the diet that are not digested or absorbed by the host into monosaccharides and short-chain fatty acids (SCFAs) [22]. SCFAs provide energy to the host and have health-promoting effects [23,24], and in the process, probiotics can maintain the dynamic balance of gut microbes. Therefore, understanding the role of probiotics in different growth stages of broilers is critical to developing effective alternatives to antibiotic growth promoters.

Our previous studies found that *B. amyloliquefaciens* TL106 can effectively protect mice against EHEC O157:H7 infection by relieving inflammation, improving intestinal barrier function, and stabilizing the gut microbiota, suggesting that it has good probiotic effects in animals and also has the potential for an in-feed antibiotic substitute [25]. In the present study, we supplemented different levels of *B. amyloliquefaciens* TL106 in broiler diets to explore the mechanism of *B. amyloliquefaciens* on broilers. The effects of TL106 on alternative antibiotics in broilers were evaluated by studying the growth performance, digestibility of nutrients, intestinal immune levels and barrier function, and microbiota composition of cecum and levels of SCFAs.

## 2. Materials and Methods

### 2.1. Bacterial Strains and Preparation

*B. amyloliquefaciens* TL106 was isolated from the faces of Tibetan pig. For experimental purposes, bacteria were grown from single colonies on Luria–Bertani (LB) plates in media (1% glucose, 1% maltose, 1% peptone, 1% tryptone, and 1% yeast extract) broth at 37 °C for 18 h to prepare seed cultures. High-density fermentation was performed in fermenter with a volume of 30 L containing 15 L medium, and the medium composition was as follows: 34 g/L maize powder, 5 g/L glucose, 5 g/L corn steep liquor powder, 40 g/L soybean meal, 0.2 g/L MnSO4, and 750 mL of seed cultures were added into the medium. The fermentation temperature was 37 °C. Dissolved oxygen concentration was autoregulated to be 30–50% by controlling the aeration rate of air and rotation rate. The pH was maintained at 7.0 by the automatic addition of 3 mol/L NaOH, and the anti-foaming agents were automatically added when foam generation occurred. Samples were collected every 7 h to measure biomass (including all bacteria and spores) and expressed as colony-forming units per milliliter (CFU/mL). After 28 h of fermentation, the concentration of bacteria was more than 1 × 10^13^ CFU/mL, and the spore rate reached more than 90%. After the fermentation was completed, the bacterial suspension was mixed with 9 kg wheat bran and then air drying for 24 h, which was the bacterial product used in this study [26,27].

### 2.2. Birds, Experimental Design and Management

All procedures used in the animal experiment were conducted under the guidance of The China Guidelines on the Welfare and Ethics of Laboratory Animals (ICS 65.020.30) issued by the Committee for the Protection and Use of Laboratory Animals of China Agricultural University. A total of 240 1-day-old Arbor Acres male broiler chickens (Beijing Huadu Broiler Company, Beijing, China) with similar initial weights (46.27 ± 0.90 g) were used. Birds were randomly allocated into five experimental groups. There were six replicates (8 birds per cage) for each treatment. Experimental treatments were labeled as follows: corn-soybean basal diet (CON), a basal diet supplemented with 75 mg/kg aureomycin (Chia Tai Group, Henan, China) (ANT), a basal diet supplemented with 7.5 × 10^8^ CFU/kg (Ba1), 2.5 × 10^9^ CFU/kg (Ba2), and 7.5 × 10^9^ CFU/kg (Ba3) *B. amyloliquefaciens* TL106. Basal diets (Table 1), without any antibiotics and growth promoters, were based on National Research Council (NRC) (1994).

Birds were vaccinated according to the routine vaccination program followed at the respective research institute (vaccinated with Newcastle disease vaccine on days 7 and 21 and inactivated with infectious bursal disease vaccine on day 14) and provided ad libitum access to feed and fresh water throughout the 42-day feeding trial. The initial room temperature was 35 °C, which was gradually reduced by 2 °C every week until 24 °C to provide a thermal comfort environment for the birds.

### 2.3. Sample Collection and Measurements

Bird body weight and feed intake were recorded on days 0, 14, 21, 28, 35, and 42 to calculate average daily gain (ADG), average daily feed intake (ADFI), and feed/gain ratio (F:G) of broilers. Fecal samples were sampled daily for 3 consecutive days from day 18 to 21 and from day 39 to 42 for apparent total tract nutrients utilization analysis.

One broiler from the Ba2 group of each replicate was slaughtered on d 14, 21, 28, 35, and 42 for collection of cecal digesta to analyze the composition and metabolites of the intestinal microbiota. The cecal digesta was snap-frozen in liquid nitrogen and then stored at −80 °C. At 21 and 42 d of age, one bird from each replicate was slaughtered rapidly for sample collection. Blood samples were collected and centrifuged at 4000× *g* for 10 min at 4 °C to obtain serum and kept at −80 °C, and middle parts of jejunal and ileal tissues were collected and kept at −80 °C for subsequent analysis. A segment of duodenum, jejunum, and ileum was flushed with PBS and fixed with 4% paraformaldehyde for intestinal morphology analysis.

### 2.4. Apparent Total Tract Nutrients Utilization

Fecal samples were collected and dried in a forced-air oven for 72 h at 65 °C. The dried fecal samples were ground through a 1-mm screen. Nutrient digestibility was calculated by the following equation: ND (%) = 1 − [(DC × FN)/(FC × DN)] × 100%. In this equation, ND represents apparent total tract nutrients utilization; DC is the content of Cr_2_O_3_ in diets (%), FN is the content of the nutrient in feces (%), FC is the content of Cr_2_O_3_ in feces (%), and DN is the content of the nutrient in diets (%). Dry matter (DM), crude protein (CP), ether extract (EE), and crude fiber (CF) in diet and excreta samples were analyzed [26]. The concentration of chromium in diets and faces was measured via an atomic absorption spectrometer (Hitachi Z-5000, Tokyo, Japan).

### 2.5. Determination of Cytokine Concentration in Tissues and Serum

Concentrations of tumor necrosis factor-α (TNF-α) and interleukin (IL-1β, IL-6, IL-8, IL-10, and IL-13) in serum, jejunum and ileal were determined according to the instructions of the ELISA kits (Nanjing Jiancheng Bioengineering Institute, China) [25].

### 2.6. RNA Isolation and Gene Expression Analysis

Total RNA was isolated from jejunum samples with HiPure Total RNA Mini Kit (Magen R4111-03, Guangzhou, China) following the manufacturer’s recommendations. Quantity and purity of RNA were assessed using the absorbance of a NanoDrop spectrophotometer (Thermo Fishier Scientific, Wilmington, NC, USA). Total RNA was reverse transcribed to complementary DNA (cDNA) using the PrimeScript^TM^ RT Master Mix (Takara, Kyoto, Japan) and then stored at −20 °C for further use.

Oligonucleotide primer sequences used for quantitative real-time PCR (qRT-PCR) are shown in Table 2. Intestinal tight junction protein expression levels, including zona occludens-1 (ZO-1), occludin, and claudin-1, were evaluated in the jejunum and ileum. Primers were designed using primer 6.0 software by searching the NCBI database to find specific sequences of the genes of interest in the corresponding species. Amplification and detection were carried out using TB Green Premix Ex Taq^TM^ II (Takara, Kyoto, Japan) and an AJ qTOWER 2.2 Real-Time PCR system (Analytik Jena AG, Jena, Germany). Glyceraldehyde-3-phosphate dehydrogenase (GAPDH) was used as an internal control, and all samples were measured in triplicate. The relative gene expression level was calculated by the 2^−∆∆Ct^ method [28].

### 2.7. Histology and Morphometric Analysis of the Intestine

Duodenum, jejunum, and ileum sections were fixed with 4% paraformaldehyde t and embedded in paraffin blocks, which were sliced and stained with hematoxylin and eosin (H&E) for histological examination. The VH and CD from each slice were determined.

### 2.8. Analysis of SCFAs in Digesta

Cecal digesta (0.50 g) were thawed in a centrifuge tube at 4 °C, and 8 mL ultra-pure water was added. Glass spheres were added to the digesta and thoroughly mixed. After ultrasonic oscillation for 20 min, followed by centrifugation at 4000× *g* for 15 min. Next, 0.16 mL of supernatant was transferred into a 10 mL centrifuge tube containing 7.84 mL ultra-pure water. The diluent was filtered through a 0.22-μm filter, and the extracted sample solution was determined by a high-performance ion chromatography analyzer (ICS-3000, Wilmington, DE, USA) for determination of SCFAs content [25].

### 2.9. Intestinal Microbiota

Total genomic DNA from cecal digesta of broilers was extracted by using E.Z.N.A Stool DNA Kit (Omega Biotek, GA, USA). The V3–V4 region of the bacterial 16S rRNA gene was amplified by PCR using the primers 338F (5′ ACTCCTACGGGAGGCAGCAG-3′) and 806R (5′-GGACTACHVGGGTWTCTAAT-3′) with the PCR reaction procedure: 95 °C for 3 min, 27 cycles of denaturing at 95 °C for 30 s, annealing at 55 °C for 30 s, extension at 72 °C for 45 s, single extension at 72 °C for 10 min (Wang et al., 2020). The PCR products were examined by electrophoresis and purified using an AxyPrep DNA Gel Extraction kit (Axygen Biosciences, CA, USA). The purified PCR products were quantified using QuantiFluor ST (Promega, Madison, WI, USA), and the library was sequenced on the Illumina HiSeq2500 platform [26].

Raw sequences were quality-filtered using Trimmomatic (version 3.29). Analysis of raw sequences was performed according to the following r criteria: (1) The reads below 50 bp after quality control were filtered, and the reads containing N bases were removed. (2) Sequences longer than 10 bp were merged in pairs. Sequences were classified into the same Operational Taxonomic Units (OTUs) by using Uparse (Uparse v7.0.1001) at 97% sequence identity [25]. Taxonomic classification at different levels of these OTU sequences was conducted with the RDP classifier (version 2.2), and the alpha-diversity was analyzed with Mothur (version 1.30.2). The 16S rRNA gene sequences have been submitted to the NCBI repository with the BioProject ID: PRJNA867507.

### 2.10. Data Analysis

Data were analyzed by one-way ANOVA for a completely randomized design, using the General Linear Model procedure (IBM SPSS software-2.0, Chicago, IL, USA). Duncan’s test was conducted to test the significant mean differences among treatments. Statistical differences were declared at *p* < 0.05, whereas a trend for a treatment effect was noted for *p* ≤ 0.10. The bacterial community in the cecal digesta samples at the phylum and family level were analyzed by Kruskal–Wallis method.

## 3. Results

### 3.1. Growth Performance

There were no adverse events during the whole experiment period. The effects of dietary antibiotics and *B. amyloliquefaciens* TL106 on the growth performance of broiler chickens are shown in Table 3. Supplementation with Ba2 and Ba3 in feeds increased ADG during the starter phase (days 1–21) compared with the CON group (*p* < 0.05). In addition, the ADFI in Ba1 and Ba3 groups were lower than that of the CON and ANT groups during the grower phase (days 22–42) (*p* < 0.05), whereas the ADFI in Ba groups during the whole study (days 1–41) were lower than CON and ANT groups (*p* < 0.05). The F:G of Ba groups during the grower phase (days 22–42) and the whole study (days 1–42) were even lower than the CON and ANT groups (*p* < 0.05).

### 3.2. Apparent Total Tract Nutrient Utilization

**Figure 1:**
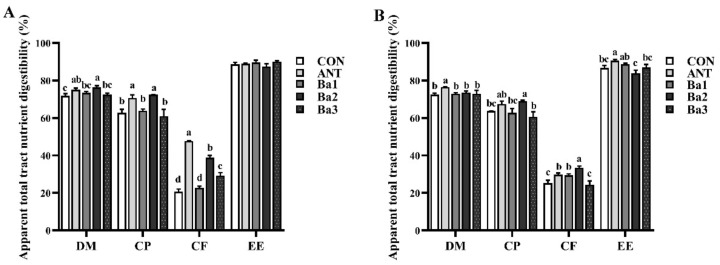
The effect of dietary antibiotics or *B. amyloliquefaciens* TL106 on apparent total tract nutrient utilization of broiler chickens. (**A**) The apparent total tract nutrient utilization of broiler chickens at day 21. (**B**) The apparent total tract nutrient utilization of broiler chickens at day 42. The data represent mean ± SEM (*n* = 3). Different lowercase letters over the bars indicate significant differences among groups (*p* < 0.05). CON, basal diet; ANT, basal diet supplemented with 75 mg/kg aureomycin; Ba1: basal diet supplemented with 7.5 × 10^8^ CFU/kg TL106; Ba2: basal diet supplemented with 2.5 × 10^9^ CFU/kg TL106; Ba3: basal diet supplemented with 7.5 × 10^9^ CFU/kg TL106.

The influence of TL106 supplementation on dietary nutrient digestibility in broiler chickens is shown in Figure 1. Compared with the CON group, the digestibility of DM and CP in ANT and Ba2 groups was higher at day 21 (*p* < 0.05). Moreover, the digestibility of CF in the ANT, Ba2, and Ba3 groups was higher compared to the CON group (*p* < 0.01) (Figure 1A). On day 42, the digestibility of DM and EE in the ANT group was higher compared to other groups (*p* < 0.05) (Figure 1B). The digestibility of CP in the Ba2 group was also significantly higher than in other groups (*p* < 0.05). Finally, the digestibility of CF in ANT, Ba1, and Ba2 groups were significantly higher than in CON and Ba3 groups, and the digestibility of CF in the Ba2 group was significantly higher than in the ANT group (*p* < 0.01) (Figure 1B).

### 3.3. Inflammatory Responses of Serum and Intestine

Concentrations of cytokines in the serum of broiler chickens are shown in Table 4. On day 21, concentrations of IL-1β and IL-6 in the ANT, Ba1, and Ba3 groups were significantly lower than in the CON group (*p* < 0.01), and concentrations of IFN-γ in the ANT and Ba1 groups were lower than CON group (*p* < 0.01). The concentration of IL-10 in the CON group was higher compared to other groups (*p* < 0.01), and the concentration of IL-13 in the ANT and Ba2 groups were lower compared to the CON group (*p* < 0.05). On day 42, compared with the CON group, concentrations of IL-1β and IL-6 in the Ba1 group were lower (*p* < 0.05), and the concentration of IL-1β was also significantly lower than in the ANT group (*p* < 0.01).

Concentrations of cytokines in the jejunum and ileum tissue of broiler chickens are shown in Table 5. In jejunal tissue, dietary supplementation with antibiotic and *B. amyloliquefaciens* TL106 decreased concentrations of IL-1β at day 21 (*p* < 0.01), in which concentrations of IL-1β in Ba2 and Ba3 groups were significantly lower than in ANT group (*p* < 0.01). On day 21, the concentration of IFN-γ in the Ba2 and Ba3 groups were lower as compared to the CON group (*p* < 0.05). The concentration of IL-6 and IL-8 in the ANT and Ba2 groups was lower compared to the CON group (*p* < 0.05). On day 42, dietary supplementation with *B. amyloliquefaciens* TL106 decreased the concentration of IL-1β and IFN-γ as compared to CON and ANT groups (*p* < 0.01). Moreover, concentrations of IL-6 and IL-8 in the Ba1, Ba2, and Ba3 groups were lower as compared to the ANT group (*p* < 0.05).

In ileum tissue, concentrations of IL-1β in dietary supplementation with antibiotic and *B. amyloliquefaciens* TL106 groups were lower compared to the CON group on day 21, and concentrations of IL-1β in Ba1 and Ba3 groups were significantly lower than ANT group (*p* < 0.01). The concentration of IFN-γ and IL-6 in *B. amyloliquefaciens* TL106-supplemented groups was significantly lower than CON group (*p* < 0.01), and the concentration of IL-6 in *B. amyloliquefaciens* TL106-supplemented groups were significantly lower than ANT group (*p* < 0.01). On day 42, the concentration of IL-1β and IFN-γ of ANT, Ba2, and Ba3 groups were lower than the CON group (*p* < 0.01). The concentration of IL-6 in antibiotic- and *B. amyloliquefaciens* TL106-supplemented groups were lower compared to the CON group (*p* < 0.01). The concentration of IL-8 and TL-10 of the ANT group was lower than the CON group (*p* < 0.05).

### 3.4. Intestinal Tight Junction Protein mRNA Expression

**Figure 2:**
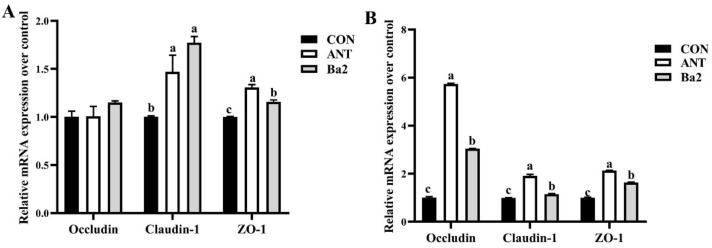
The effect of dietary antibiotics or *B. amyloliquefaciens* TL106 on the expressions of intestinal jejunal tight junction protein genes in broiler chickens. (**A**) The expressions of intestinal jejunal tight junction protein genes in broiler chickens at day 21. (**B**) The expressions of intestinal jejunal tight junction protein genes in broiler chickens at day 42. The data represent mean ± SEM (*n* = 3). Different lowercase letters over the bars indicate significant differences among groups (*p* < 0.05). Transcript levels of the tight junction protein were measured using quantitative RT-PCR and normalized to GAPDH transcript levels. CON, basal diet; ANT, basal diet supplemented with 75 mg/kg aureomycin; Ba2: basal diet supplemented with 2.5 × 10^9^ CFU/kg TL106.

The expression of jejunal tight junction protein genes detected in the different groups is shown in Figure 2. On day 21, the expression of genes claudin-1 and ZO-1 of ANT and Ba groups were significantly higher compared to the CON group (*p* < 0.01) (Figure 2A). On day 42, the expression of genes occludin, claudin-1, and ZO-1 of ANT and Ba groups were higher than CON group (*p* < 0.01) (Figure 2B), and the expression of genes occludin and claudin-1 of Ba group were lower than ANT group, while expression of gene ZO-1 was higher than ANT group (*p* < 0.01) (Figure 2B).

### 3.5. Intestinal Morphology

Effects of dietary treatment on intestinal morphology at days 21 and 42 are shown in Figure S1 and Table 6. On day 21, dietary supplementation with antibiotic and *B. amyloliquefaciens* TL106 increased VH of the duodenum (*p* < 0.01), and CD of ANT and Ba3 groups were higher than the CON group (*p* < 0.01). The duodenal VH/CD ratio of the Ba1 and Ba2 groups was higher than the CON group (*p* < 0.01). The jejunal VH of the Ba2 and Ba3 groups were higher than CON and ANT groups (*p* < 0.01), and the VH/CD ratio of the Ba2 group was higher than CON and ANT groups (*p* < 0.01). In the ileum, dietary supplementation with *B. amyloliquefaciens* TL106 tended to influence ileal VH/CD (*p* = 0.059). On day 42, the duodenal VH and VH/CD ratio of the Ba3 group was lower than other groups (*p* < 0.05), and the duodenal CD of the Ba1 group was lower than CON and ANT groups (*p* < 0.01). Dietary probiotics supplementation did not affect (*p* > 0.05) the intestinal parameters in the jejunum and ileum of broilers on day 42.

### 3.6. SCFAs of Cecal Digesta

On day 14, the concentrations of lactate, acetate, and propionate in cecal digesta of the Ba2 group were higher than those of the CON group (*p* < 0.01), while the concentrations of formate, isobutyrate, isovalerate, and valerate in cecal digesta of the Ba2 and ANT groups were lower as compared to CON group (*p* < 0.01) (Table 7).

On day 21, the concentrations of lactate, formate, and valerate in the cecal digesta of the Ba2 group were higher than CON group (*p* < 0.05), while the concentrations of valerate in the cecal digesta of the Ba2 group were higher than in ANT group (*p* < 0.01). Additionally, the concentrations of isobutyrate, butyrate, and isovalerate were significantly higher in the ANT group than CON group (*p* < 0.05).

On day 28, the increased concentrations of propionate, butyrate, and valerate in the cecal digesta of the Ba2 group were higher than CON group (*p* < 0.05), and the concentrations of propionate and butyrate were also higher than in the ANT group (*p* < 0.05). The concentration of lactate in the Ba2 group was lower than CON and ANT groups (*p* < 0.01).

On day 35, the concentrations of lactate, formate, butyrate, isovalerate, and valerate in cecal digesta of the Ba2 group were lower as compared to the CON group (*p* < 0.05), and the concentrations of lactate, butyrate, and valerate were also lower than those of ANT group (*p* < 0.01).

On day 42, the Ba2 group was higher than the concentrations of propionate, formate, and valerate in cecal digesta as compared to the CON group (*p* < 0.05), while the concentrations of propionate and formate were higher than the ANT group (*p* < 0.05). In the ANT group, the concentrations of lactate and butyrate were higher than group (*p* < 0.05). In addition, the concentrations of isobutyrate and isovalerate were higher in the cecal digesta of the CON group than in the ANT and Ba2 groups (*p* < 0.05).

### 3.7. Bacterial Diversities and Community Compositions

α diversity index could reflect the richness and diversity of bacterial communities. The Sobs, ACE, and Chao indexes reflected the bacterial community richness, and the Shannon and Simpson indexes reflected the bacterial community diversity. Where the greater the value of the Simpson index, the lower the community diversity. The Sobs, ACE, and Chao indexes of cecal digesta of broilers in ANT and Ba2 groups were higher than those in the CON group during the starter phase (days 1–21). Similarly, in the starter phase (days 1–21), the Shannon index of cecal digesta of broilers in the ANT and Ba2 groups was higher than that in the CON group, while the Simpson index was lower than that in the CON group (Table S1).

**Figure 3:**
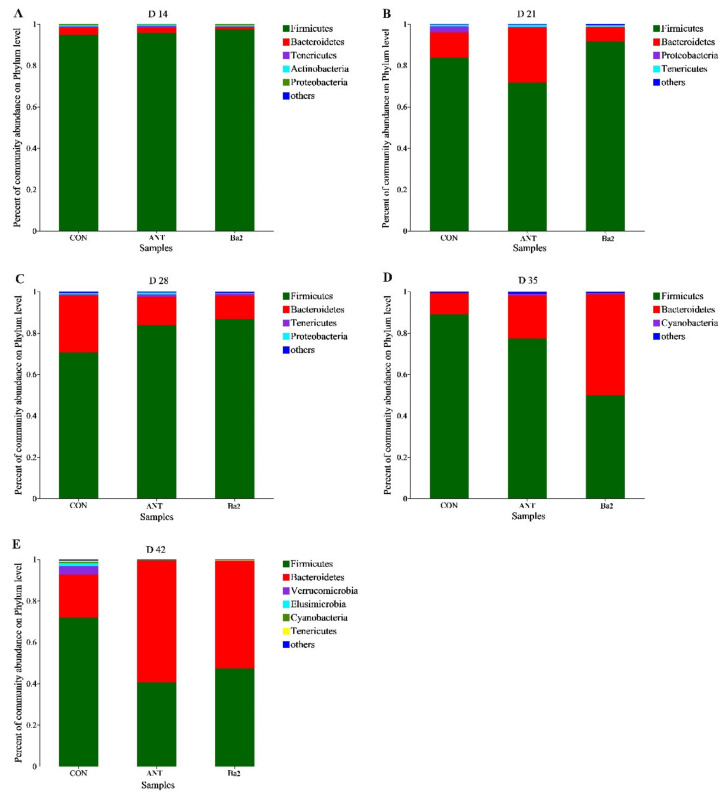
The effect of *B. amyloliquefaciens* TL106 on gut microbiota composition at phylum levels with a relative abundance higher than 0.005%. (**A**) Microbial community on the phylum level on day 14. (**B**) Microbial community on the phylum level on day 21. (**C**) Microbial community on the phylum level on day 28. (**D**) Microbial community on the phylum level on day 35. (**E**) Microbial community on the phylum level on day 42. CON, basal diet; ANT, basal diet supplemented with 75 mg/kg aureomycin; Ba2: basal diet supplemented with 2.5 × 10^9^ CFU/kg TL106.

The bacterial communities at the phylum level in the cecum are shown in Figure 3. At 14 days, there was no difference in the abundance of microbiota between all groups. The percentage of *Firmicutes* was higher in the Ba2 group than in the control group on 21 and 28 days. Moreover, dietary supplementation with antibiotics and *B. amyloliquefaciens* TL106 reduced the percentage of *Proteobacteria* as compared with the control group in 21 days. At 35 and 42 days, the percentage of *Bacteroidetes* was higher in the ANT and Ba2 groups than the CON group, and the relative proportions of *Firmicutes* and *Bacteroidetes* were decreased in the ANT and Ba2 groups than the control group. In addition, the increase in the total relative abundance of *Firmicutes* and *Bacteroides* in ANT and Ba2 groups resulted in a decrease in the abundance of the rest phyla on 42 days.

**Figure 4:**
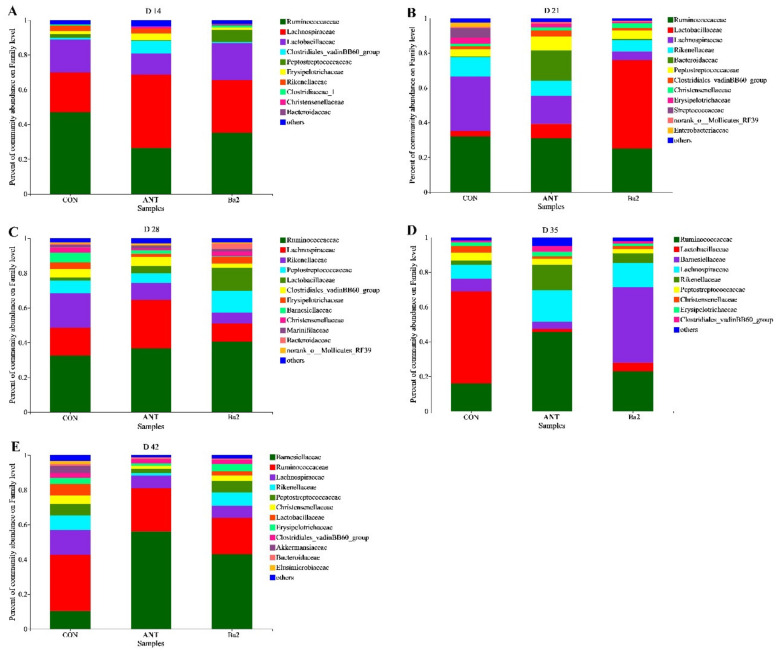
The effect of *B. amyloliquefaciens* TL106 on gut microbiota composition at family levels with a relative abundance higher than 0.01%. (**A**) Microbial community on the family level on day 14. (**B**) Microbial community on the family level on day 21. (**C**) Microbial community on the family level on day 28. (**D**) Microbial community on the family level on day 35. (**E**) Microbial community on the family level on day 42. CON, basal diet; ANT, basal diet supplemented with 75 mg/kg aureomycin; Ba2: basal diet supplemented with 2.5 × 10^9^ CFU/kg TL106.

The bacterial communities at the family level in the cecum were analyzed (Figure 4). The percentage of *Peptostreptococcaceae* was higher in the Ba2 group than in the control group on 14, 21, and 28 days. At 21 days, dietary supplementation with *B. amyloliquefaciens* TL106 increased the abundance of *Lactobacillaceae* and decreased the percentage of *Lachnospiraceae*, *Rikenellaceae*, *Erysipelotrichaceae,* and *Enterobacteriaceae* compared control group. At 28 days, the abundance of *Ruminococcaceae* and *Lactobacillaceae* increased in the Ba2 group compared with the control group. The abundance of *Rikenellaceae* and *Clostridiales-vadinBB60-group* was decreased in the Ba2 group. At 35 days, dietary supplementation with *B. amyloliquefaciens* TL106 improved the abundance of *Ruminococcaceae*, *Barnesiellaceae*, *Lachnospiraceae*, and *Rikenellaceae* compared with the control group but decreased the abundance of *Lactobacillaceae*. At 42 days, dietary supplementation with *B. amyloliquefaciens* TL106 improved the abundance of *Barnesiellaceae* and *Peptostreptococcaceae* compared with the control group, but decreased the abundance of *Ruminococcaceae*, *Lachnospiraceae*, *Christensenellaceae*, and *Lactobacillaceae*.

The composition of cecal microflora obviously changed with time. From days 14 to 42, the percentage of the phylum of *Firmicutes* gradually decreased, but on days 14 to 28, the percentage in the *Bacillus* group was higher than the control group, and on days 35 and 42, the proportion in the *Bacillus* group was lower than the control group. However, the percentage of phylum of *Bacteroidetes* gradually increased, and even at 35 and 42 days, the percentage of *Bacteroidetes* in the antibiotic and *Bacillus* groups was higher than control group.

## 4. Discussion

Due to the beneficial function of promoting animal growth and regulating intestinal microbial homeostasis in animals, *Bacillus* has been widely used in the field of animal production as a feed additive [29,30,31]. Previous studies indicate that the addition of *Bacillus* as a probiotic in broiler diets can improve growth performance [11,32]. In this study, the administration of *B. amyloliquefaciens* TL106 into diets resulted in a decrease in ADFI and F:G of broilers during the whole study (days 1–42). In addition, ADFI and F:G of broilers supplemented with *B. amyloliquefaciens* TL106 were significantly lower than that of broilers supplemented with antibiotics (75 mg/kg). These results indicate that *B. amyloliquefaciens* TL106 can improve the feed conversion rate of broilers more effectively than antibiotics with a concentration of 75 mg/kg, implying that *B. amyloliquefaciens* TL106 has the potential to replace antibiotics in poultry growth promotion. The phenotype that the addition of *B. amyloliquefaciens* can improve the growth performance of broilers was shaped synthetically by multifaceted effects, such as immune response ability, VH and CD of the gut, intestinal barrier function, and the development and composition of intestinal microbiota. On the one hand, probiotics can promote intestinal development and improve the capacity for nutrient absorption in animals [33]. On the other hand, probiotics can also regulate the composition of intestinal microflora, further promoting the intestinal micro ecosystem balance [10].

*Bacillus* exerts prebiotic effects by promoting gut health through multiple-faceted mechanisms of action [11]. An important characteristic of *Bacillus* is that they can produce various digestive enzymes such as amylases, proteases, cellulases, and lipases [34]. These enzymes can enhance the digestion and absorption of nutrients in the digestive tract. The *B. amyloliquefaciens* also secrete a variety of bioactive substances such as antimicrobial proteins, lipopeptides, etc., which are able to inhibit the growth of harmful bacteria, promote the multiplication of beneficial bacteria, and maintain intestinal homeostasis [11,35].

In the present study, supplemented with *B. amyloliquefaciens* TL106 in broiler diets significantly increased the apparent digestibility of DM, CP, and CF compared to the control group, with the digestibility of CF even higher than the group supplemented with antibiotics. These results indicate that *B. amyloliquefaciens* can effectively improve the apparent total tract digestibility of CP and CF. In addition, *Bacillus* promoted further improvements in nutrient digestion and absorption over time to a greater extent than antibiotic inclusion. This was in accordance with the findings of Tejeda and Kim [36], who suggested that with *Bacillus* supplementation in broiler diet, the improvement of broilers’ growth performance was associated with better nutrient digestibility. Similarly, a report on the improvement of broiler performance with the addition of *B. amyloliquefaciens* to the diet was associated with an increase in the digestibility of nutrients [37].

Subsequently, dietary supplementation with *Bacillus* can increase the digestibility of nutrients, which is closely related to intestinal health and intestinal development. Both VH and CD and their ratios are important indicators of intestinal digestive properties, which directly reflect the absorption capacity of intestinal mucosa [38]. The crypts have a secretory function, and CD can reflect the rate of regeneration of intestinal epithelial cells [39]. In the current study, *B. amyloliquefaciens* TL106 increased the VH or VH/CD ratio of duodenum and jejunum compared with the control group on day 21. When the supplemental level of *B. amyloliquefaciens* TL106 in the broiler diet was 7.5 × 10^8^ CFU/kg, there was a decrease in CD of the duodenum. These results indicate that supplementation with *B. amyloliquefaciens* TL106 can improve the intestinal structure of the duodenum and jejunum and further improve the absorptive surface. The above results might also explain some of the reasons for the improvement of nutrient digestibility and growth performance when TL106 is added to the broiler diet. Similarly, an increase in VH and VH/CD ratio was observed in broilers supplemented with B. subtilis or *B. amyloliquefaciens* [40,41].

Previous studies have found that cytokines are associated with intestinal mucosal inflammation and can be used for intestinal disease assessment [42]. Pathogens stimulate intestinal epithelial cells to produce pro-inflammatory cytokines such as IL-6 and IL-8, which recruit immune cells to the inflammatory site [43]. These immune cells will produce more pro-inflammatory cytokines such as IL-1β and IFN-γ, thereby damaging intestinal health and increasing intestinal epithelial permeability [44]. Additionally, IL-10 and IL-13 have anti-inflammatory effects and are crucial in the control of immune responses and intestinal health [45]. In this study, *B. amyloliquefaciens* treatment decreased the concentration of pro-inflammatory cytokines (IL-1β, IFN-γ, IL-6, and IL-8) in the serum, jejunum, and ileum. Similar decreases in IL-6 in intestinal epithelial lymphocytes of chickens provided diets supplemented with various *B. subtilis* strains were reported [46].

The intestinal epithelium is an important part of intestinal mucosal immunity, and the tight junction barrier plays an important role in preventing invasion by pathogens, endotoxins, and feed-associated antigens [47,48]. Tight junction protein is a protein complex that maintains the integrity of the intestinal epithelial barrier by sealing adjacent epithelial cells [49]. Decrease in tight junction protein expression results in impaired gut barrier function, accompanied by increased intestinal permeability [25]. TJs mainly include occludin, claudin, JAM, and tricellulin, which interact with cytoplasmic scaffold proteins (ZO) [50]. Claudin-1 can effectively prevent harmful substances from reaching the surface of epithelial cells [51], and high expression of claudin-1 could lead to increased epithelial compactness and decreased intestinal permeability [52]. Occludin helps regulate paracellular permeability and plays a key role in cell structure and barrier function. ZO has multiple domains, which can provide corresponding binding sites for transmembrane proteins and promote the formation of tight junction protein skeleton. At the same time, ZO can bind to each other, making the structure of tight junction protein skeleton more stable Bazzoni et al. and Gadde et al. [53,54] reported that oral administration of *Bacillus subtilis* improved the protein levels of occludin and ZO-1 in the small intestine of broilers. Similar to these results, we observed that oral administration of *B. amyloliquefaciens* upregulated the expression of tight junction protein occludin, claudin-1, and ZO-1 in the jejunum. The level of TJ protein expression was increased after the addition of *B. amyloliquefaciens* TL106 to the broiler diet, which could promote the enhancement of intestinal barrier function and gut health.

The gut microbiota plays an important role in maintaining the integrity of the gastrointestinal barrier [55]. In the present study, the core phyla of the cecal microbiota were mainly *Firmicutes* and *Bacteroidetes*, regardless of different treatments or different phases of broilers. This result was consistent with previous studies in broilers [56], laying hens [24], and geese [57]. In our study, in addition to *Firmicutes* and *Bacteroidetes*, *Proteobacteria* and *Tenericutes* contributed to the core phyla of cecal microbiota from 0 to 28 days. However, from 29 to 42 days, *Cyanobacteria*, *Verrucobacteria*, and *Elusimicrobia* were added to the core phyla indicating that the diversity of gut microbes increases with age. The changes in the core phyla in the intestinal microbiota of broilers over time were consistent with previous findings, and these changes are due to the physiological needs of birds [58,59].

The results of this study showed a greater *Firmicutes* to *Bacteroidetes* ratio and total overall subsets in Firmicutes and *Bacteroidetes* in broilers provided antibiotic and TL106-supplemented diets. Studies have shown that *Firmicutes* in the gut of animals are positively correlated with the ability to obtain energy from feed [60], and the ratio of *Firmicutes* to *Bacteroidetes* may also have important effects on animal physiology and nutrition [61]. Based on these considerations, the addition of T106 or antibiotics in broiler diets appears to better modulate the gut microbiota. In the present study, the relative abundances of *Peptostreptococcaceae* and *Lactobacillaceae*, which belong to the *Firmicutes* phylum, were especially dramatic on days 21 and 28. *Peptostreptococcaceae* is an important symbiotic bacterial family, and its relative abundance in healthy people was higher than that in those with intestinal flora imbalance, suggesting that *Peptostreptococcaceae* has the potential to maintain the stability of the intestinal environment [62,63]. *Lactobacillus* have a strong ability to adhere to epithelial tissues, can inhibit the colonization of poor bacteria, and have a bacteriostatic effect [64]. The results of the present study showed that the relative abundance of *Lactobacillus* in the cecal digesta of broilers fed *B. amyloliquefaciens* TL106 was higher than the control group. *Lactobacillus* are the main producers of lactic acid [65], which may explain the increased lactic acid levels in the groups with the addition of antibiotics and *B. amyloliquefaciens* TL106. Increased levels of SCFAs produced by microbial metabolism of the animal gut may lower the pH of the gut, which in turn creates an environment that is not conducive to the growth of harmful microorganisms, promoting nutrient digestion and absorption by the host [24]. On days 35 and 42 of this study, relative abundance of *Bacteroidetes* increased, while the relative abundance of *Firmicutes* decreased. Many members of the *Bacteroidetes* contribute to the digestion and absorption of nutrients in the host gut [66] and can ferment carbohydrates in the intestinal tract and produce SCFAs. Therefore, the abundance of *Bacteroidetes* is also affected by dietary components [67]. Compared with the control group, the abundance of *Barnesiellaceae*, a family belonging to the phylum *Bacteroidetes*, in the Ba group of the cecal digesta was significantly higher. *Barnesiellaceae* family has been shown to play a crucial role in maintaining physical health and catabolizing sugars [68]. In addition, *Barnesiellaceae* is an effective producer of SCFAs, particularly butyrate and propionate [69,70], a major energy source for colonocytes that protect the intestinal barrier. Previous studies have shown that adding *Bacillus subtilis* to diets can improve the relative abundance of *Barnesiellaceae* in the cecum of broilers [71]. Similarly, the results of the present study showed that dietary supplementation of *B. amyloliquefaciens* TL106 can improve the relative abundance of *Barnesiellaceae* in the cecum of broilers on days 35 and 42. In summary, supplementation of *B. amyloliquefaciens* TL106 in the diet of broilers modulated the development and composition of the gut microbiota of broilers and promoted microbial metabolism to produce volatile fatty acids, which in turn promoted intestinal development and intestinal health.

## 5. Conclusions

In conclusion, supplementation of *B. amyloliquefaciens* TL106 to the diets of broilers can improve growth performance, immune and anti-inflammatory levels of serum and intestinal tissues, intestinal morphology, and expression of tight junction protein-related genes in broilers. In addition, dietary supplementation TL106 can also regulate the cecal microbial composition and SCFAs production in broilers. Our results suggest that *B. amyloliquefaciens* TL106 may be an alternative to in-feed antibiotics to promote growth and metabolism in broilers.

## Figures and Tables

**Table 1:** Composition and nutrient levels of basal diets (as-fed basis, %).

Item	Starter (Day 1–21)	Finisher (Day 22–42)
Ingredients		
Corn, 8.5% crude protein	61.51	67.99
Soybean meal, 44% crude protein	28.00	22.00
Fish meal, 64.6% crude protein	3.71	2.60
Soybean oil	3.00	3.60
Dicalcium phosphate, 22% calcium	1.28	1.38
Limestone, 38% calcium	1.26	1.10
Salt	0.30	0.30
_L_-Lysine HCL, 98%	0.00	0.12
Methionine, 98%	0.15	0.07
Threonine, 98%	0.04	0.09
Tryptophan, 98%	0.00	0.00
Chromic oxide	0.25	0.25
Vitamin-mineral premix ^a^	0.50	0.50
Total	100.00	100.00
Nutrient levels		
Metabolizable energy, kcal/kg	3052.68	3157.48
Crude protein	20.08	17.37
Calcium	1.00	0.90
Available phosphorus	0.45	0.42
Lysine	1.10	1.00
Methionine	0.50	0.38

^a^ Vitamin-mineral premix provided the following per kilogram of diet: vitamin A, 9000 IU; vitamin D3, 3000 IU; vitamin E, 24 mg; vitamin K3, 1.8 mg; thiamine, 2.0 mg; riboflavin, 5.0 mg; pyridoxine, 3.0 mg; cobalamin, 0.1 mg; nicotinic acid, 40 mg; pantothenic acid, 15 mg; folic acid, 1.0 mg; biotin, 0.05 mg; choline chloride, 500 mg; Fe, 80 mg as iron sulfate; Cu, 20 mg as copper sulfate; Zn, 90 mg as zinc sulfate; Mn, 80 mg as manganese sulfate; I, 0.35 mg as potassium iodide; Se, 0.35 mg as sodium selenite.

**Table 2:** Oligonucleotide primer sequences for qRT-PCR.

Target Gene	Primer Sequence (5′-3′)	PCR Product Size (Kb)
*ZO-1*	F-CTTCAGGTGTTTCTCTTCCTCCTC	131
	R-CTGTGGTTTCATGGCTGGATC	
*Occludin*	F-ACGGCAGCACCTACCTCAA	123
	R-GGGCGAAGAAGCAGATGAG	
*Claudin-1*	F-CATACTCCTGGGTCTGGTTGGT	100
	R-GACAGCCATCCGCATCTTCT	
*GAPDH*	F-TGCTGCCCAGAACATCATCC	142
	R-ACGGCAGGTCAGGTCAACAA	

**Table 3:** Effect of dietary antibiotics or *B. amyloliquefaciens* TL106 on growth performance of broiler chickens.

Item	Treatments					SEM	*p*-Value
	CON	ANT	Ba1	Ba2	Ba3		
Body weight							
Day 1	46.47	46.13	46.22	46.22	46.32	0.17	0.980
Day 21	609.84	613.62	620.77	631.84	625.04	4.14	0.484
Day 42	1902.22	2023.87	2079.22	2049.72	2049.38	22.59	0.100
Day 1–21							
ADG (g)	25.93 ^b^	27.03 ^ab^	27.35 ^ab^	28.24 ^a^	27.59 ^a^	0.25	0.040
ADFI (g)	33.48	35.02	33.45	33.29	32.24	0.61	0.739
F:G	1.29	1.30	1.22	1.18	1.17	0.02	0.191
Day 22–42							
ADG (g)	61.24	65.68	67.50	66.95	67.44	0.80	0.055
ADFI (g)	107.44 ^a^	108.19 ^a^	96.22 ^b^	101.60 ^ab^	95.08 ^b^	1.82	0.048
F:G	1.75 ^a^	1.65 ^ab^	1.42 ^c^	1.52 b ^c^	1.41 ^c^	0.03	0.000
Day 1–42							
ADG (g)	44.18	47.09	48.40	47.71	47.70	0.54	0.098
ADFI (g)	71.75 ^a^	73.25 ^a^	64.83 ^b^	65.59 ^b^	63.24 ^b^	1.14	0.006
F:G	1.62 ^a^	1.55 ^a^	1.34 ^b^	1.38 ^b^	1.33 ^b^	0.03	0.000

^a–c^ Within the same row, values with different superscripts mean significant difference (*p* < 0.05). CON, basal diet; ANT, basal diet supplemented with 75 mg/kg aureomycin; Ba1: basal diet supplemented with 7.5 × 10^8^ CFU/kg TL106; Ba2: basal diet supplemented with 2.5 × 10^9^ CFU/kg TL106; Ba3: basal diet supplemented with 7.5 × 10^9^ CFU/kg TL106; ADG: average daily gain; ADFI: average daily feed intake; F:G: feed-to-gain ratio; SEM: standard error of the mean.

**Table 4:** Effect of dietary antibiotics or *B. amyloliquefaciens* TL106 on concentration of cytokines (pg/mL) in serum of broiler chickens.

Item	Treatments					SEM	*p*-Value
	Control	ANT	Ba1	Ba2	Ba3		
Day 21							
IL-1β	67.31 ^a^	61.60 ^c^	59.53 ^c^	66.41 ^ab^	63.25 ^bc^	0.89	0.004
IFN-γ	9.21 ^a^	7.29 ^b^	7.75 ^b^	9.38 ^a^	8.68 ^a^	0.24	0.001
IL-6	27.44 ^a^	23.37 ^b^	24.01 ^b^	27.76 ^a^	25.07 ^b^	0.55	0.004
IL-8	14.57	13.17	14.50	14.37	14.11	0.28	0.562
IL-10	8.95 ^a^	7.09 ^b^	7.80 ^b^	7.86 ^b^	7.43 ^b^	0.19	0.005
IL-13	5.81 ^a^	4.99 ^b^	5.80 ^a^	5.45 ^b^	5.94 ^a^	0.12	0.029
Day 42							
IL-1β	64.70 ^b^	76.51 ^a^	58.67 ^c^	63.34 ^b^	65.37 ^b^	1.65	0.000
IFN-γ	8.46	7.54	7.67	9.49	9.06	0.28	0.091
IL-6	27.42 ^a^	26.27 ^ab^	23.45 ^b^	29.52 ^a^	27.57 ^a^	0.68	0.034
IL-8	13.20	13.88	14.04	14.89	14.91	0.27	0.198
IL-10	7.57	6.48	8.29	8.63	8.40	0.28	0.077
IL-13	4.98	5.15	5.41	6.06	5.60	0.15	0.054

^a–c^ Within the same row, values with different superscripts mean significant difference (*p* < 0.05). CON, basal diet; ANT, basal diet supplemented with 75 mg/kg aureomycin; Ba1: basal diet supplemented with 7.5 × 10^8^ CFU/kg TL106; Ba2: basal diet supplemented with 2.5 × 10^9^ CFU/kg TL106; Ba3: basal diet supplemented with 7.5 × 10^9^ CFU/kg TL106; SEM: standard error of the mean.

**Table 5:** Effect of dietary antibiotics or *B. amyloliquefaciens* TL106 on concentration of cytokines (pg/mg) in jejunum and ileum tissue of broiler chickens.

Item		Treatments					SEM	*p*-Value
		CON	ANT	Ba1	Ba2	Ba3		
Day 21								
Jejunum	IL-1β	59.84 ^a^	43.72 ^c^	51.83 ^b^	33.88 ^d^	34.00 ^d^	2.67	0.000
	IFN-γ	8.47 ^a^	6.62 ^ab^	7.25 ^ab^	5.62 ^bc^	3.96 ^c^	0.48	0.018
	IL-6	24.44 ^a^	12.87 ^c^	22.87 ^ab^	17.73 ^bc^	19.44 ^ab^	1.30	0.010
	IL-8	8.26 ^a^	4.54 ^c^	7.74 ^ab^	6.04 ^bc^	6.72 ^ab^	0.42	0.010
	IL-10	8.45	7.56	6.43	6.29	5.92	0.38	0.191
	IL-13	4.74	3.19	4.09	3.58	3.72	0.26	0.421
Ileum	IL-1β	71.07 ^a^	48.39 ^b^	25.33 ^c^	40.19 ^b^	27.54 ^c^	4.60	0.000
	IFN-γ	8.47 ^a^	7.33 ^ab^	4.06 ^d^	5.57 ^cd^	6.09 ^bc^	0.45	0.001
	IL-6	31.22 ^a^	31.58 ^a^	16.07 ^c^	21.16 ^b^	14.73 ^c^	1.98	0.000
	IL-8	8.52 ^abc^	11.00 ^a^	8.03 ^bc^	7.48 ^c^	10.37 ^ab^	0.47	0.042
	IL-10	7.51 ^a^	6.68 ^ab^	3.97 ^d^	6.16 ^b^	5.14 ^c^	0.35	0.000
	IL-13	6.18 ^a^	4.45 ^b^	2.53 ^d^	4.00 ^bc^	3.51 ^c^	0.34	0.000
Day 42								
Jejunum	IL-1β	43.67 ^b^	51.55 ^a^	35.40 ^c^	26.14 ^d^	30.33 ^d^	2.52	0.000
	IFN-γ	6.61 ^a^	6.59 ^a^	5.25 ^b^	4.58 ^bc^	4.27 ^c^	0.28	0.000
	IL-6	20.89 ^b^	26.00 ^a^	21.00 ^b^	9.78 ^c^	18.12 ^b^	1.51	0.000
	IL-8	7.62 ^ab^	9.42 ^a^	5.80 ^b^	6.71 ^b^	7.07 ^b^	0.42	0.046
	IL-10	6.10	7.44	6.05	8.61	6.36	0.38	0.136
	IL-13	3.63	4.62	4.67	4.47	3.96	0.17	0.195
Ileum	IL-1β	32.75 ^a^	24.40 ^c^	34.14 ^a^	28.33 ^b^	29.41 ^b^	0.97	0.000
	IFN-γ	6.03 ^a^	3.75 ^c^	5.29 ^ab^	4.70 ^bc^	4.30 ^bc^	0.24	0.003
	IL-6	18.16 ^a^	9.79 ^c^	14.05 ^b^	14.43 ^b^	11.38 ^c^	0.82	0.000
	IL-8	8.44 ^a^	4.93 ^b^	7.78 ^a^	6.90 ^a^	6.92 ^a^	0.38	0.015
	IL-10	4.43 ^a^	2.88 ^b^	4.41 ^a^	3.34 ^ab^	4.07 ^a^	0.21	0.032
	IL-13	3.33	2.61	3.39	3.56	3.11	0.14	0.279

^a–c^ Within the same row, values with different superscripts mean significant difference (*p* < 0.05). CON, basal diet; ANT, basal diet supplemented with 75 mg/kg aureomycin; Ba1: basal diet supplemented with 7.5 × 10^8^ CFU/kg TL106; Ba2: basal diet supplemented with 2.5 × 10^9^ CFU/kg TL106; Ba3: basal diet supplemented with 7.5 × 10^9^ CFU/kg TL106; SEM: standard error of the mean.

**Table 6:** Effect of dietary antibiotics or *B. amyloliquefaciens* TL106 on intestinal morphology of broiler chickens on the age of days 21 and 42.

Item		Treatments					SEM	*p*-Value
		CON	ANT	Ba1	Ba2	Ba3		
Day 21								
Duodenum	VH, μm	827.74 ^b^	1081.57 ^a^	1150.21 ^a^	1082.00 ^a^	1152.43 ^a^	35.67	0.001
	CD, μm	111.98 ^b^	126.66 ^a^	110.04 ^b^	110.77 ^b^	132.54 ^a^	2.90	0.005
	VH/CD	7.40 ^c^	8.52 ^bc^	10.52 ^a^	9.78 ^ab^	8.71 ^bc^	0.33	0.003
Jejunum	VH, μm	447.22 ^b^	523.24 ^b^	538.08 ^b^	760.88 ^a^	760.20 ^a^	36.99	0.000
	CD, μm	70.20 ^b^	66.77 ^b^	74.28 ^b^	75.26 ^b^	95.30 ^a^	2.86	0.000
	VH/CD	6.36 ^b^	7.90 ^b^	7.30 ^b^	10.10 ^a^	7.99 ^b^	0.39	0.008
Ileum	VH, μm	375.48	355.21	439.09	414.62	549.28	24.94	0.084
	CD, μm	75.38	77.35	91.60	67.62	70.79	2.93	0.057
	VH/CD	5.02	4.61	4.73	6.18	8.01	0.45	0.059
Day 42								
Duodenum	VH, μm	1456.95 ^a^	1528.50 ^a^	1472.91 ^a^	1636.38 ^a^	1178.18 ^b^	51.47	0.032
	CD, μm	135.74 ^c^	153.10 ^b^	118.97 ^d^	155.57 ^b^	181.57 ^a^	5.93	0.000
	VH/CD	10.82 ^a^	10.03 ^a^	12.39 ^a^	10.53 ^a^	6.53 ^b^	0.59	0.004
Jejunum	VH, μm	836.86	907.91	888.37	733.81	848.04	30.43	0.461
	CD, μm	98.67	110.62	99.63	93.52	102.02	2.93	0.510
	VH/CD	8.51	8.37	9.03	7.83	8.25	0.29	0.827
Ileum	VH, μm	582.11	638.17	709.15	707.17	649.39	22.39	0.376
	CD, μm	102.97	106.55	94.31	108.49	114.33	3.30	0.446
	VH/CD	5.66	6.01	7.79	6.52	5.79	0.37	0.402

^a–d^ Within the same row, values with different superscripts mean significant difference (*p* < 0.05). CON, basal diet; ANT, basal diet supplemented with 75 mg/kg aureomycin; Ba1: basal diet supplemented with 7.5 × 10^8^ CFU/kg TL106; Ba2: basal diet supplemented with 2.5 × 10^9^ CFU/kg TL106; Ba3: basal diet supplemented with 7.5 × 10^9^ CFU/kg TL106; VH: the villus height; CD: crypt depth; VH/CD: the ratio of villus height to crypt depth; SEM: standard error of the mean.

**Table 7:** Effect of dietary antibiotics or *B. amyloliquefaciens* TL106 on the concentrations of cecal digesta SCFAs (mg/kg) in broiler chickens during different phases.

Items	Lactate	Acetate	Propionate	Formate	Isobutyrate	Butyrate	Isovalerate	Valerate
Day 14								
CON	464.83 ^b^	2880.70 ^c^	281.24 ^c^	90.72 ^a^	265.01 ^a^	927.57	23.24 ^a^	87.40 ^a^
ANT	59.42 ^c^	3721.36 ^b^	402.44 ^b^	23.98 ^b^	39.62 ^b^	934.08	7.45 ^c^	9.81 ^c^
Ba2	1066.05 ^a^	5207.66 ^a^	633.31 ^a^	38.52 ^b^	47.30 ^b^	1005.53	10.62 ^b^	39.60 ^b^
SEM	147.42	352.94	53.34	10.41	37.78	17.28	2.42	11.36
*p*-value	0.000	0.000	0.000	0.000	0.000	0.109	0.000	0.000
Day 21								
CON	2125.21 ^b^	3137.77 ^b^	504.41 ^a^	47.07 ^b^	31.37 ^b^	606.55 ^b^	6.03 ^b^	19.77 ^c^
ANT	9549.71 ^a^	3695.46 ^ab^	716.32 ^b^	29.01 ^a^	51.04 ^a^	720.71 ^a^	28.04 ^a^	31.43 ^b^
Ba2	9472.29 ^a^	4592.67 ^a^	811.48 ^c^	19.63 ^a^	36.33 ^ab^	62.44 ^c^	4.08 ^b^	85.35 ^a^
SEM	1258.93	253.10	47.74	4.14	3.65	102.71	3.94	10.12
*p*-value	0.000	0.027	0.001	0.000	0.042	0.000	0.000	0.000
Day 28								
CON	65.92 ^b^	2681.98	512.28 ^b^	16.55	51.83	340.43 ^c^	14.45 ^a^	4.02 ^c^
ANT	87.40 ^a^	2742.87	448.61 ^b^	16.37	39.87	418.68 ^b^	7.01 ^b^	26.87 ^a^
Ba2	51.57 ^c^	2323.15	654.82 ^a^	19.48	43.56	533.20 ^a^	17.11 ^a^	23.33 ^b^
SEM	5.36	118.59	35.09	0.93	2.24	29.63	1.66	3.59
*p*-value	0.000	0.336	0.015	0.350	0.054	0.001	0.005	0.000
Day 35								
CON	52.75 ^a^	4032.45 ^a^	611.42	63.07 ^a^	78.93 ^a^	1044.89 ^a^	52.10 ^a^	51.28 ^a^
ANT	48.44 ^a^	2515.81 ^b^	605.57	21.83 ^b^	50.00 ^b^	864.44 ^a^	14.25 ^b^	52.18 ^a^
Ba2	32.06 ^b^	3203.50 ^ab^	682.99	27.00 ^b^	89.63 ^a^	675.87 ^b^	13.08 ^b^	28.82 ^b^
SEM	3.54	253.41	18.47	6.75	6.20	59.32	6.48	4.11
*p*-value	0.009	0.016	0.163	0.000	0.001	0.007	0.000	0.003
Day 42								
CON	51.19 ^b^	4241.43	851.58 ^b^	11.39 ^b^	123.52 ^a^	984.63 ^b^	43.08 ^a^	42.08 ^b^
ANT	103.76 ^a^	5372.42	789.00 ^b^	12.35 ^b^	82.58 ^b^	1331.25 ^a^	25.37 ^b^	82.49 ^a^
Ba2	63.03 ^b^	5095.13	1117.65 ^a^	19.00 ^a^	70.63 ^b^	1178.46 ^ab^	22.54 ^b^	80.67 ^a^
SEM	8.24	268.64	59.71	1.46	8.22	58.60	3.47	6.97
*p*-value	0.000	0.215	0.024	0.034	0.000	0.019	0.003	0.001

^a–c^ Within the same row, values with different superscripts mean significant difference (*p* < 0.05). CON, basal diet; ANT, basal diet supplemented with 75 mg/kg aureomycin; Ba2: basal diet supplemented with 2.5 × 109 CFU/kg TL106; SEM: standard error of the mean.

## Data Availability

Sequencing data in this study has been submitted to the NCBI repository with the BioProject ID: PRJNA867507. Additional data are available upon request.

## Supplementary Materials

The following supporting information can be downloaded at: https://www.mdpi.com/article/10.3390/ani12223085/s1, Figure S1. Representative histopathology section of intestines. (A) Sliced duodenal tissue of 21-day-old broilers from each group was stained by H&E staining and was observed under a DM3000 microscope (Leica Microsystems, Wetzlar, Germany) with 40 × original magnification. (B) Sliced duodenal tissue of 42-day-old broilers. (C) Sliced jejunal tissue of 21-day-old broilers. (D) Sliced jejunal tissue of 42-day-old broilers. (E) Sliced ileal tissue of 21-day-old broilers. (F) Sliced ileal tissue of 42-day-old broilers. CON, basal diet; ANT, basal diet supplemented with 75 mg/kg aureomycin; Ba1: basal diet supplemented with 7.5 × 10^8^ CFU/kg TL106; Ba2: basal diet supplemented with 2.5 × 10^9^ CFU/kg TL106; Ba3: basal diet supplemented with 7.5 × 10^9^ CFU/kg TL106. Table S1. Effects of *B. amyloliquefaciens* TL106 on alpha-diversity of cecal bacterial community in broilers. Alpha-diversity analysis for bacterial community determined by 16s RNA sequencing. Sobs are the number of observed operational taxonomic units (OTUs). CON, basal diet; ANT, basal diet supplemented with 75 mg/kg aureomycin; Ba2: basal diet supplemented with 2.5 × 10^9^ CFU/kg TL106.
