# Supplementary material for: The Effects of Dietary Bacillus amyloliquefaciens TL106 Supplementation, as an Alternative to Antibiotics, on Growth Performance, Intestinal Immunity, Epithelial Barrier Integrity, and Intestinal Microbiota in Broilers
Source: Animals (Basel). 2022 Nov 9;12(22):3085. doi: 10.3390/ani12223085 (PMC9686771; doi:10.3390/ani12223085)
Supplement: Supplementary file 1 [file animals-12-03085-s001.zip › animals-1986218-supplementary.pdf]

animals

Supplementary Materials

The Effects of Dietary *Bacillus amyloliquefaciens* TL106 Supplementation, as an Alternative to Antibiotics, on Growth Performance, Intestinal Immunity, Epithelial Barrier Integrity, and Intestinal Microbiota in Broilers

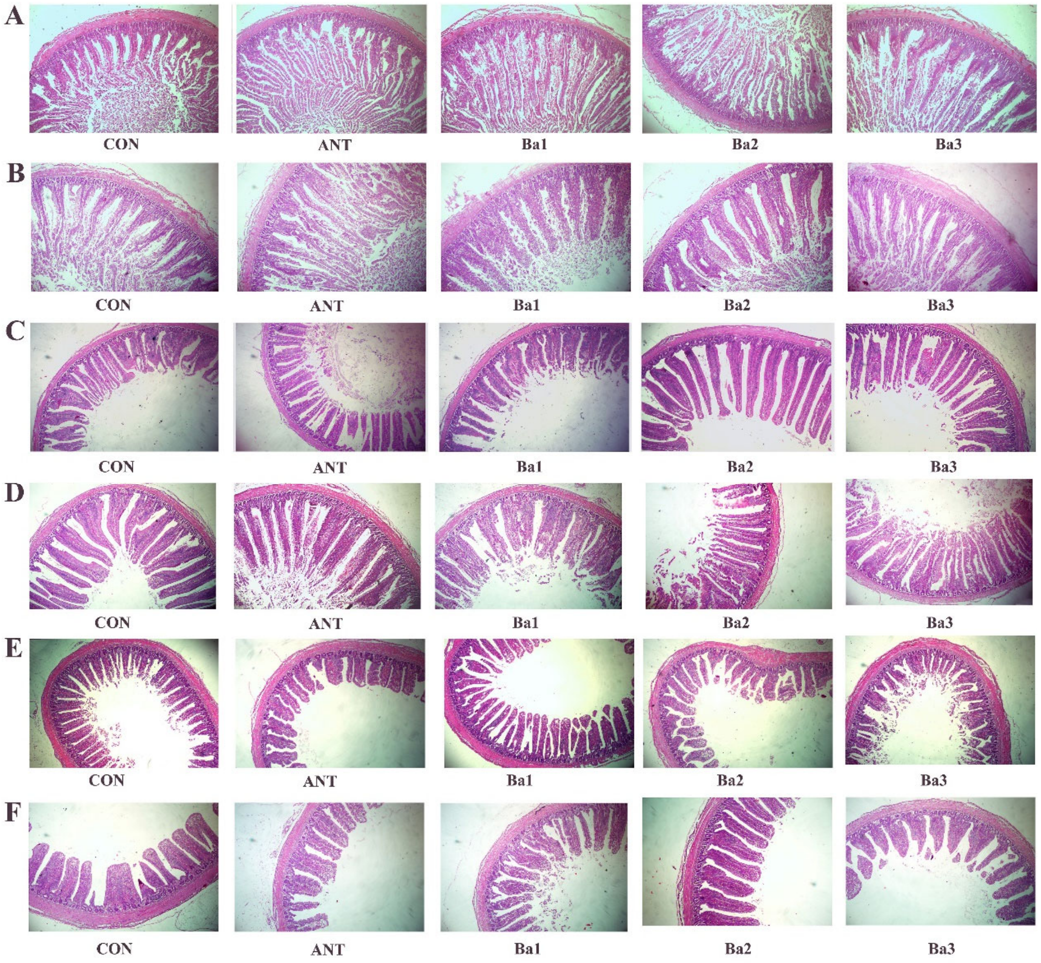

Figure S1. Representative histopathology section of intestines. (A) Sliced duodenal tissue of 21-day-old broilers from each group was stained by H&E staining and was observed under a DM3000 microscope (Leica Microsystems, Wetzlar, Germany) with 40 × original magnification. (B) Sliced duodenal tissue of 42-day-old broilers. (C) Sliced jejunal tissue of 21-day-old broilers. (D) Sliced jejunal tissue of 42-day-old broilers. (E) Sliced ileal tissue of 21-day-old broilers. (F) Sliced ileal tissue of 42-day-old broilers. CON, basal diet; ANT, basal diet supplemented with 75 mg/kg aureomycin; Ba1: basal diet supplemented with 7.5×10^{8} CFU/kg TL106; Ba2: basal diet supplemented with 2.5×10^{9} CFU/kg TL106; Ba3: basal diet supplemented with 7.5×10^{9} CFU/kg TL106.

Table S1. Effects of *B. amyloliquefaciens* TL106 on alpha-diversity of cecal bacterial community in broilers[1].

Items	Sobs	Shannon	Simpson	Ace	Chao
**Day 14**					
CON	230	3.43	0.06	247.90	247.55
ANT	316	4.32	0.03	338.41	337.57
Ba2	291	3.58	0.06	323.91	324.44
**Day 21**					
CON	322	3.09	0.13	374.80	379.78
ANT	444	4.24	0.03	497.77	511.53
Ba2	389	3.97	0.04	433.65	427.89
**Day 28**					
CON	474	4.27	0.03	535.64	553.07
ANT	465	4.21	0.04	510.26	507.90
Ba2	462	4.10	0.04	513.78	525.55
**Day 35**					
CON	449	3.18	0.16	546.60	552.06
ANT	527	4.28	0.04	579.10	588.13
Ba2	454	3.48	0.13	529.30	526.23
**Day 42**					
CON	526	4.42	0.02	567.43	566.25
ANT	494	3.41	0.14	564.65	568.75
Ba2	427	2.55	0.30	513.53	537.53

[1]alpha-diversity analysis for bacterial community determined by 16s RNA sequencing. Sobs are the number of observed operational taxonomic units (OTUs). CON, basal diet; ANT, basal diet supplemented with 75 mg/kg aureomycin; Ba2: basal diet supplemented with 2.5×10^9 CFU/kg TL106.
